# Synovial Fluid Progenitors Expressing CD90+ from Normal but Not Osteoarthritic Joints Undergo Chondrogenic Differentiation without Micro-Mass Culture

**DOI:** 10.1371/journal.pone.0043616

**Published:** 2012-08-29

**Authors:** Roman J. Krawetz, Yiru Elizabeth Wu, Liam Martin, Jerome B. Rattner, John R. Matyas, David A. Hart

**Affiliations:** 1 Department of Surgery, University of Calgary, Calgary, Alberta, Canada; 2 Department of Cell Biology and Anatomy, University of Calgary, Calgary, Alberta, Canada; 3 Department of Medicine, University of Calgary, Calgary, Alberta, Canada; 4 Department of Comparative Biology and Experimental Medicine, Faculty of Veterinary Medicine, University of Calgary, Calgary, Alberta, Canada; Instituto Butantan, Brazil

## Abstract

**Objective:**

Mesenchymal progenitor cells (MPCs) can differentiate into osteoblasts, adipocytes, and chondrocytes, and are in part responsible for maintaining tissue integrity. Recently, a progenitor cell population has been found within the synovial fluid that shares many similarities with bone marrow MPCs. These synovial fluid MPCs (sfMPCs) share the ability to differentiate into bone and fat, with a bias for cartilage differentiation. In this study, sfMPCs were isolated from human and canine synovial fluid collected from normal individuals and those with osteoarthritis (human: clinician-diagnosed, canine: experimental) to compare the differentiation potential of CD90+ vs. CD90− sfMPCs, and to determine if CD90 (Thy-1) is a predictive marker of synovial fluid progenitors with chondrogenic capacity *in vitro*.

**Methods:**

sfMPCs were derived from synovial fluid from normal and OA knee joints. These cells were induced to differentiate into chondrocytes and analyzed using quantitative PCR, immunofluorescence, and electron microscopy.

**Results:**

The CD90+ subpopulation of sfMPCs had increased chondrogenic potential compared to the CD90− population. Furthermore, sfMPCs derived from healthy joints did not require a micro-mass step for efficient chondrogenesis. Whereas sfMPCs from OA synovial fluid retain the ability to undergo chondrogenic differentiation, they require micro-mass culture conditions.

**Conclusions:**

Overall, this study has demonstrated an increased chondrogenic potential within the CD90+ fraction of human and canine sfMPCs and that this population of cells derived from healthy normal joints do not require a micro-mass step for efficient chondrogenesis, while sfMPCs obtained from OA knee joints do not differentiate efficiently into chondrocytes without the micro-mass procedure. These results reveal a fundamental shift in the chondrogenic ability of cells isolated from arthritic joint fluids, and we speculate that the mechanism behind this change of cell behavior is exposure to the altered milieu of the OA joint fluid, which will be examined in further studies.

## Introduction

Within the last decade, the synovium and synovial fluid have been identified as a source of mesenchymal progenitor/stem cells (sMPCs) that are functionally distinct from bone marrow MPCs [1]–[7]. Furthermore, these synovial membrane (smMPCs) and sfMPCs can assist in cartilage repair both *in vivo* and *in vitro* [8]–[11]. There is compelling evidence that these cells can be used effectively to promote cartilage repair [1]–[11]. sfMPCs reportedly have increased chondrogenic potential [12], [13] compared to bone marrow-derived mesenchymal stem cells (bmMPCs), including increased expression of the hyaluronan receptor CD44 [13], [14] and uridine diphosphoglucose dehydrogenase (UDPGD), an enzyme required for hyaluronan synthesis. Notably, bmMPCs and other MPC populations do not express UDPGD [13], [14]. Evidence from *in vivo* studies have demonstrated that when partial-thickness defects in the articular cartilage of rabbits are formed, a continuous cell layer extending from the synovial membrane is observed to contribute to the repair of the cartilage either with or without chondrogenic inducers present [9], [10]. In addition, pig and human sfMPCs have been transformed *in vitro* into scaffolds termed Tissue Engineering Constructs (TECs) that can be used to repair cartilaginous defects (in pigs) *in vivo* [6], [7]. Additionally, another group has recently demonstrated that smMPCs have the ability to bind directly to cartilage *ex vivo* in minutes, and are able to contribute to cartilage repair in a defect model [11]. Human sfMPCs are typically characterized using cluster of differentiation (CD) antigens [15]: CD105 (Endoglin), CD90 (Thy-1), CD73 (Ecto-5′-nucleotidase) and CD44 are present on the surface of MPCs/MSCs, while CD45 (Protein tyrosine phosphatase, receptor type, C) and CD11b (Integrin alpha M) are not expressed by this cell population [15]. The present study focuses on CD90 (Thy-1), which has been shown to interact with Integrins, tyrosine kinases, growth factors, and cytokines thereby promoting downstream cellular events including: adhesion, apoptosis, proliferation, and migration [16]. CD90 is commonly used as a marker of MPCs/MSCs, though it is also expressed by many other cell types including neurons, endothelial cells, T-cells, and other immune/non-immune cell types [16]. More recently, CD90 has been utilized as a selection marker of multi-potent progenitors from bone marrow, synovial tissues, fat, amnion and other tissues [17]. However, the exact role of CD90 on the surface of this class of cells remains unknown.

A number of recent studies have begun to explore the role of sfMPCs in diseases, including arthritis. Initial reports suggested that there was no difference in the chondrogenic potential of sfMPCs derived from healthy joints and joints with osteoarthritis (OA) or rheumatoid arthritis (RA) [2], notwithstanding the increase in number of sfMPCs in the OA knees [2]. A more recent study by the same group reported that the inflammatory intra-articular environment in RA joints is responsible for the reduced chondrogenic potential of sfMPCs [18]. As OA is generally viewed primarily as a degenerative rather than an inflammatory joint disease, it seems that the milieu of RA and OA joints has a fundamentally different influence on the capacity of sfMPCs to proliferate and differentiate. If, as has been speculated, sfMPCs participate in processes of joint maintenance or repair after injury [9], [10], a fuller understanding of sfMPCs is warranted as they are potential therapeutic targets for these common and debilitating joint diseases.

In a recent study where synovial membrane stem cells were obtained from OA patients and differentiated using a micro-mass tissue culture, a significant positive correlation was observed between CD90 expression and chondrogenic differentiation [19]. Therefore, the aim of the present study is a comparison of the chondrogenic potential of sfMPCs (human and canine) isolated from normal and osteoarthritic synovial fluid.

## Results

### Differentiation potential of normal and OA derived sfMPCs

**Figure 1:**
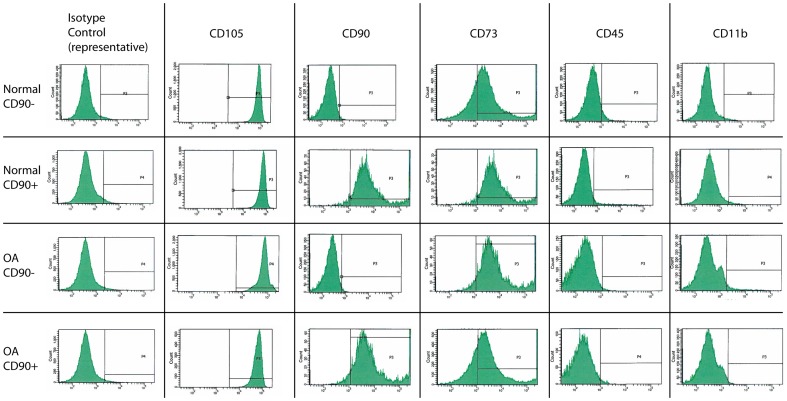
Micro-mass differentiation of sfMPCs. Normal (human N = 5 A–D & canine N = 2 H–K) and OA (human N = 5 E–G & canine N = 2 L–N) derived sfMPCs where aggregated using the micro-mass technique and induced to differentiate over a 14 day period with media supplements. qRT-PCR results demonstrated that Sox9, Collagen 2 and Aggrecan are significantly elevated at days 3, 5, 8 and 14 of differentiation (A,E,H,L), furthermore, at day 14 Collagen 2 protein is expressed in the cell cultures (B,F,I,M). Secondary controls (C,J) demonstrate minimal non-specific staining. Scale bars represent 50 µM. * = p>0.05.

To evaluate the chondrogenic potential of human sfMPCs (CD105+, CD73+, CD44+, CD45−. CD11b−) and canine sfMPCs (CD45−, CD34−) derived from normal and OA synovial fluid, the cells were differentiated into chondrocytes with media supplements over a 14 day period with a prior micro-mass aggregation step. At days 0, 3, 5, 8 and 14, mRNA was collected and probed using qRT-PCR for Sox9, Collagen 2, and Aggrecan (Figure 1 A,E,H,L). By day 14, Sox9, Collagen 2 and Aggrecan were significantly elevated compared to day 0 controls in normal (Figure 1 A,E) and OA (Figure 1 H,L) sfMPCs derived from human and canine synovial fluid. Immunofluorescence confirmed the qRT-PCR data using a Collagen 2 antibody on day 14. sfMPC-derived chondrogenic masses from normal (Figure 1 B,F) and OA (Figure 1 I,M) fluid expressed Collagen 2 protein on day 14. Secondary antibody alone controls demonstrated minimal non-specific staining in human (Figure 1 C) and canine (Figure 1 J) sfMPCs. Furthermore, the micro-masses generated from all conditions stained with Alcian blue (Figure 1 D,G,K,N).

### Enhanced Chondrogenic Differentiation of CD90+ sfMPCs

**Figure 2:**
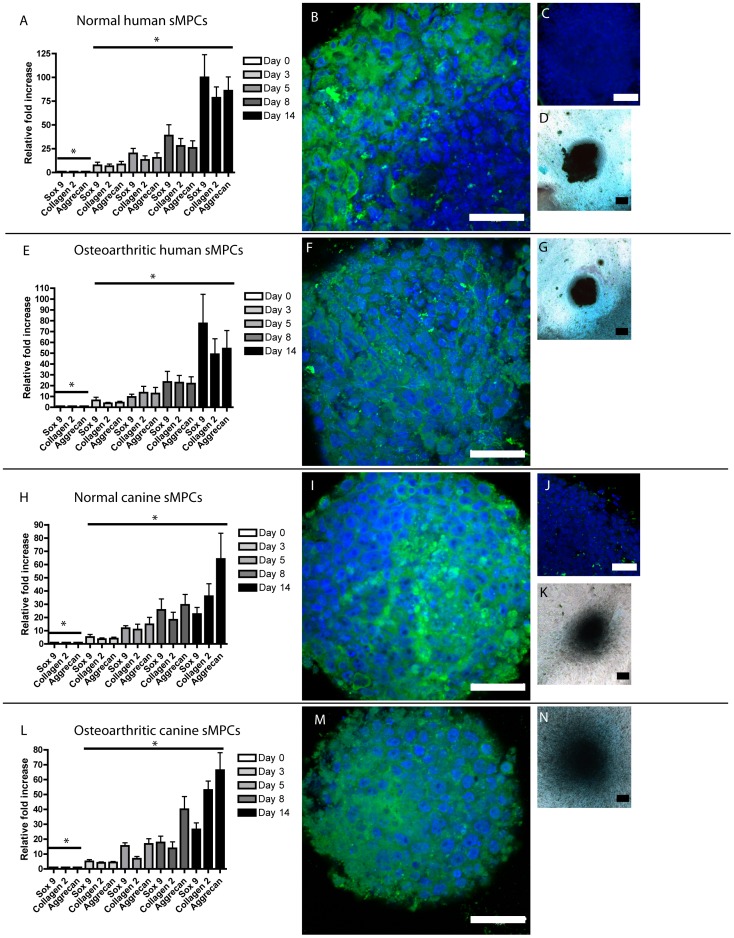
Chondrogenic Differentiation of CD90+/− sfMPCs. sfMPCs derived from normal synovial fluid were enriched/depleted for CD90 and induced to differentiate into chondrocytes. The CD90+ fraction expressed (A,F,K,P) similar levels of Sox9, Collagen 2 and Aggrecan mRNA on day 14 to the total sMPC population (Figure 2), whereas the CD90− fraction demonstrated reduced levels of Sox9, Collagen 2 and Aggrecan (A,F,K,P). Alcian blue (D,I,N,S) and Collagen 2 (E,J,O,T) staining intensity was also decreased in the CD90− negative fraction compared to the CD90+ fraction (Alcian blue: B,G,L,Q, Collagen 2:C,H,M,R) on day 14. Scale bars represent 250 µM.

Since the human and canine sfMPCs contained CD90 positive and negative cells, the chondrogenic potential of the CD90-positive and CD90-negative fractions within the sfMPC population were studied. Previously isolated sfMPCs (Figure 2) were further enriched for CD90 using immuno-magnetic separation, with the resultant CD90-positive and CD90-negative fractions induced to differentiate into chondrocytes utilizing a micro-mass step (Figure 2). CD90+ sfMPCs from normal individuals (human and canine) behaved in a similar fashion to the total sfMPC population, displaying significantly increased levels of Sox9, type II Collagen, and Aggrecan mRNA (Figure 2 A,K) and demonstrated positive staining for Alcian blue (Figure 2 B,L) and Collagen 2 (Figure 2 C,M) by day 14. The CD90-negative fraction displayed reduced levels of Sox9, Collagen 2 and Aggrecan mRNA (although significantly increased compared to day 0)(Figure 2 A,K), as well as less intense alcian blue (Figure 2 D,N) and Collagen 2 (Figure 2 E, O) staining at day 14 of differentiation compared to the CD90+ population. Similarly, human and canine CD90+ sfMPCs from OA fluid demonstrated increased Sox9, Collagen 2 and Aggrecan mRNA expression (Figure 2 F,P), as well as staining with alcian blue (Figure 2 G,Q) and Collagen 2 (Figure 2 H,R) compared to the CD90− sfMPCs. Specifically, the mRNA level (Figure 2 F,P) and staining with alcian blue (Figure 2 I,S) and Collagen 2 (Figure 2 J,T) were decreased in the CD90− sfMPCs compared to the CD90+ sfMPCs.

**Figure 3:**
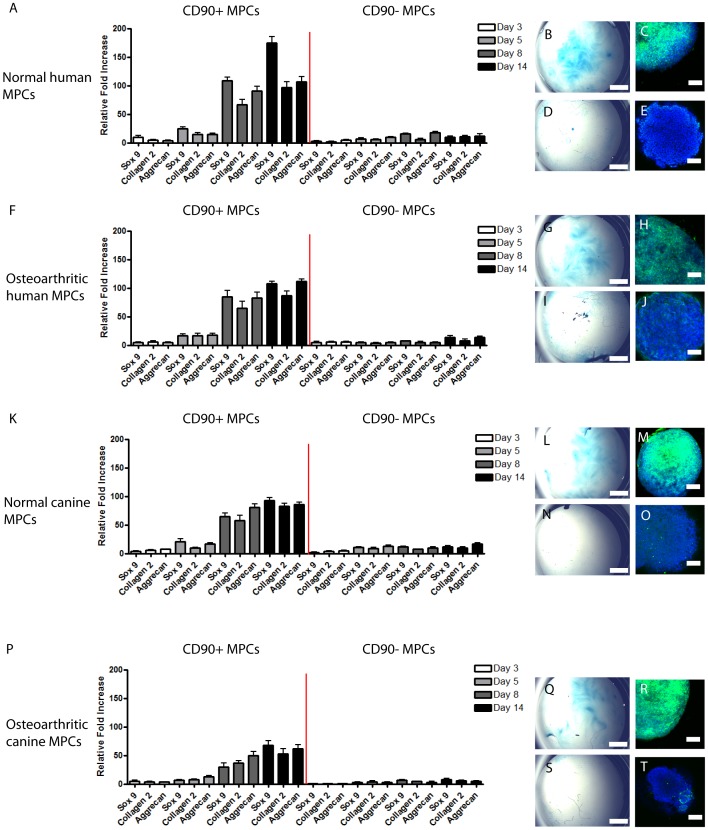
Multi-potent differentiation of CD90+/− sfMPCs. Normal and OA CD90+ or CD90− sfMPCs were induced to differentiate into osteoblasts or adipocytes and stained with Akaline phosphatase (A) to indentify osteoblasts, or Oil Red-O (B) to identify lipid-containing cells (adipocytes). qRT-PCR was used to determine the relative level of adipocyte specific genes (Adiponectin, PPAR-gamma, [C]) and osteoblastic specific genes (Osteonectin, Sp7/Osterix [D]). * = p>0.05.

To examine if there were any differences in the osteogenic or adipogenic capacity of CD90+/− human sfMPCs (normal & OA), the cells were placed in adipogenic or osteogenic conditions and allowed to differentiate for 20 days (Figure 3). After 20 days of differentiation, the cells were stained for Alkaline phosphatase (Figure 3 A) or Oil Red-O (Figure 3 B), and assayed using qPCR for Adiponectin, PPAR-gamma, Osteonectin and Osterix (Sp7) (Figure 3 C–F). Using these outcome measures, no differences in osteogenic or adipogenic differentiation capacity were observed between normal and OA sfMPCs, or CD90+/− sub-populations of either group.

### Chondrogenesis of normal CD90-positive sfMPCs without a prior micro-mass step

**Figure 4:**
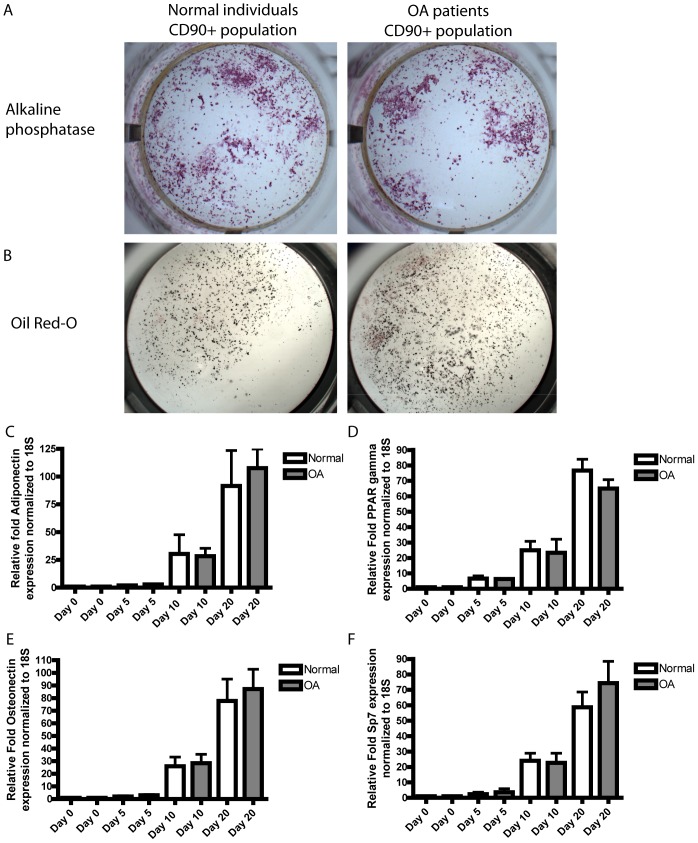
Aggregation and Chondrogenesis of sfMPCs. Normal and OA CD90+ sfMPCs were induced to differentiate without prior micro-mass aggregation. By day 14 normal sfMPCs expressed Sox9, Collagen2 and Aggrecan mRNA (A,D) while OA sfMPCs expressed severely reduced levels (A,D). Furthermore, normal sfMPCs generated a tissue-like structure that stained positive for Alcian Blue (B,G), whereas OA sfMPCs did not (C,F) and remained as a monolayer. Scale bars represent 200 µM.

Based on the foregoing results, the chondrogenic potential of normal and OA sfMPSc were studied without micro-mass culture. Without a prior micro-mass step, normal sfMPCs from human (Figure 4 A) and canine (Figure 4 D) up-regulated Sox 9, Collagen 2 and Aggrecan mRNA during differentiation. In contrast, the Sox 9, Collagen 2 and Aggrecan mNRA levels in OA sfMPCs were significantly lower (Figure 4 A,D). Normal (human and canine) sfMPCs aggregated into small tissue-like structures during differentiation (Figure 4 B,G); OA-derived sfMPCs (human and canine) did not aggregate (Figure 4 C,F).

### CD90-positive Cells Form a Tissue-like Structure

**Figure 5:**
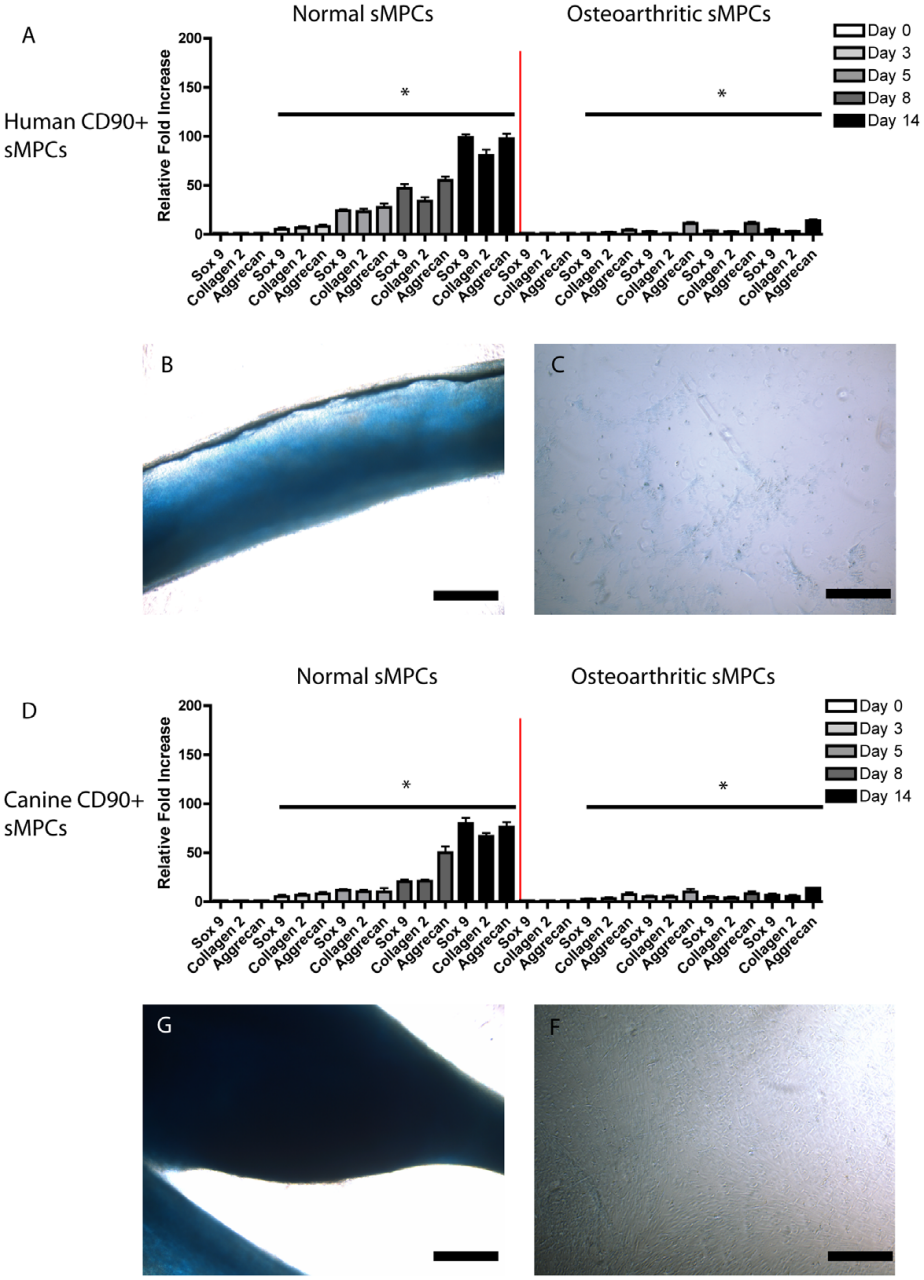
Staining and TEM of Derived Tissue from Normal sfMPCs. The resultant tissue-like masses were stained with antibodies to Collagen 2 (A) and Aggrecan (C), nuclei were stained with TOTO3 (B, D). Cells were found in closer association to each other (E), with higher magnification showing production of collagen fibres (F). Cells were also found in proximity with the produced collagen ECM (E, F). Scale bars represent 200 µM.

The tissue-like structures formed by normal CD90+ sfMPCs stained positively with antibodies against Aggrecan (Figure 5 A) and Collagen 2 (Figure 5 C). When visualized with TEM, these tissue-like structures were highly cellular (Figure 5 E). TEM analysis demonstrated that the cells were in close association with each other (Figure 5 F), and were also in proximity to collagen fibres (Figure 5 G). The cells in these self-assembled structures were attached to the surrounding collagen fibre network of the extracellular matrix (Figure 5 E and F).

## Discussion

OA is a degenerative disorder of synovial joints characterized by articular cartilage degradation and meager evidence of repair or regeneration. Although evidence of cartilage stem/progenitor cells has been difficult to obtain, which may explain this response, recent animal studies have raised the possibility that MPCs present in the synovial membrane or joint fluid may play a role in repair cartilage *in vivo* [9], [10].

MPCs are of particular interest to regenerative medicine as they have the ability to generate bone, cartilage, and muscle under certain conditions. Indeed, properly harnessed, these MPCs could be utilized *in-vivo* to promote the repair or replacement of tissue damaged by trauma or disease. MPCs for skeletal regeneration are most commonly sourced from the bone marrow or fat, and it is increasing recognized that many adult tissues have resident stem cell populations. Although seemingly the ideal source of MPCs for cartilage regeneration, it has been claimed that adult articular cartilage uniquely lacks ‘true’ MPCs [21]. Interestingly, MPC populations have been isolated from the synovial membrane and synovial fluid bathing the joint [14]. Moreover, when compared to MPC populations isolated from bone marrow or fat [12], sfMPCs demonstrate an increased chondrogenic capacity compared to MPC populations isolated from bone marrow or fat [12].

This current study demonstrates that normal and OA (human and canine) derived sfMPCs undergo effective chondrogenesis when a micro-mass step is included in the differentiation protocol, which corroborates previously published findings [2]. Furthermore, when sfMPCs (normal/OA) are enriched for the CD90-positive fraction, enhanced chondrogenesis is observed compared to the CD90-depleted fraction. This is of particular interest as a recent study by Nagase et al. reports that the chondrogenic potential of adult stem cells decrease with the subsequent loss of CD90 [19], while another study seems to contradict this observation [22]. The results of the present study supports those of Nagase et al. suggesting that CD90 is a cell surface marker that correlates positively with chondrogenic potential [19]. It is also noteworthy that patients with even advanced OA have sfMPCs, and in some cases with numbers far exceeding normal individuals [2], which could be interpreted as a cellular repair response.

A particularly noteworthy finding in the present study is that CD90-positive sfMPCs (normal/OA) can differentiate into chondrocytes without a micro-mass step. However, whereas whole populations of sfMPCs from normal individuals aggregate and express high levels of chondrogenic markers, whole populations of sfMPCs from OA joints do not aggregate spontaneously, grow only as a monolayer, and express low levels of chondrogenic markers during differentiation. The exact functional significance of micromass aggregation is unclear, yet micro-mass-induced aggregation clearly increases the efficiency of chondrogenesis [23], [24], possibly by mimicking embryonic tissue condensation. Although the promotion of cell differentiation by aggregation is not limited to sfMPCs and chondrogenesis, (having been demonstrated with pancreatic, hepatic, fibroblastic, bone marrow, and cord blood MPCs [25]–[30]), it seems curious that all normal and OA sfMPCs behave differently in non-aggregating culture even though they were otherwise derived and cultured under identical conditions. Moreover, it would appear that this aggregation behaviour (which was consistent across the 5 human and 2 canine normal individuals tested) is lost within the OA phenotype (again consistent across the 5 OA patients and 2 canines tested). We speculate that MPCs in the OA joint might be missing, or have masked, a cell surface receptor necessary for spontaneous aggregation. In continuing studies, we will examine if OA sfMPCs demonstrate any changes in gene expression compared to normal sfMPCs, specifically in genes related to pre-condensation (CCN-1, CNN-2) and/or within condensation (N-CAM, Tenascin C) [31]. To complement this line of investigation, micro-array studies comparing the global gene expression patterns of normal vs. OA sfMPCs have been completed and the data analysis is underway. Previously, studies have demonstrated that chondrocyte aggregation is mediated by β1-Integrin [32], while Cadherins seem to play a dominant role in the aggregation of MSCs during chondrogenesis [33]. Therefore, the mechanism behind the failure of OA sfMPCs aggregation should be studied further to determine if genes expressed during the normal chondrogenic developmental process are aberrantly expressed.

Since OA leads to a progressive loss of cartilage and synovial progenitors cells have the potential to contribute to articular cartilage repair *in vivo* [34], the inability of OA sfMPCs to spontaneously differentiate into chondrocytes suggests that cell-to-cell aggregation and/or communication may be impaired in OA and somehow dampen the normal mechanism of chondrocyte replenishment from the synovium or synovial fluid. Should the cells of the synovium or synovial fluid be a reservoir of stem cells for normal articular cartilage maintenance and repair, these endogenous sources of chondro-biased cells would be a fundamental and new strategy for treating OA and cartilage injury if this loss of aggregation & differentiation phenotype can be overcome.

## Methods

### Ethics Statements

Human: Informed consent to participate was obtained by written agreement. The study protocol was approved by the University of Calgary Research Ethics Board (Application number: 21987).

Canine: All procedures received approval by the institutional ethics board (University of Calgary, Animal Care Committee) and were carried out under the supervision of a veterinarian according to the guidelines of the Canadian Council on Animal Care.

### Human Subjects

Patients with clinical and radiographic OA [20] with no other co-morbidities consented to arthrocentesis and had synovial fluid aspirated from their knees (mean volume 63.4 ml+/−12.6 ml) during a visit to the University of Calgary Foothills Medical Clinic (N = 5, 3 males age: 47, 50, 52; 2 females age 52, 54; Ethics ID #21987). Synovial fluid from macroscopically normal knees was aspirated (mean volume 5.6 ml+/−2.2 ml) from cadavers less than 4 hrs after death (N = 5, 2 males age: 47, 54; 3 female age 46, 51, 52 Ethics ID #21987). Tissue donors were received by the Southern Alberta Organ and Tissue Donation Program (SAOTDP), which obtains the medical history of every donor, including current medication, previous history of joint diseases, and other co-morbidities (e.g., cancer, diabetes, inflammatory diseases). All donor knees received x-ray and macroscopic examination of the joint surfaces. Any abnormalities (cracking, blistering, darkening, abnormal wear) prompted exclusion from the study. In total seven cadaveric joints were examined and two were excluded from the study.

### Animals

Animals were adult, outbred, skeletally mature canines ranging in age from 18–54 months. Briefly, unilateral cruciate ligament transection was performed through a lateral peripatellar athrotomy (N = 2, 1 male, 1 female). Eight weeks after surgery, animals were euthanized, and sterile samples of joint fluid were withdrawn with a syringe. The volume of joint fluid from un-operated control knee joints ranged from 150–300 micro-liters; the volume of joint fluid from knees with cruciate transection ranged from 5–8 ml. Joint fluid was kept at 4°C and transported to the laboratory within 2 hours of necropsy. All knee and hip joints were inspected for morphological abnormalities, which were not observed in any of the ipsilateral or contralateral hips, nor in any of the ipsilateral (control) knee joints.

### sMPC derivation and purification

**Figure 6:**
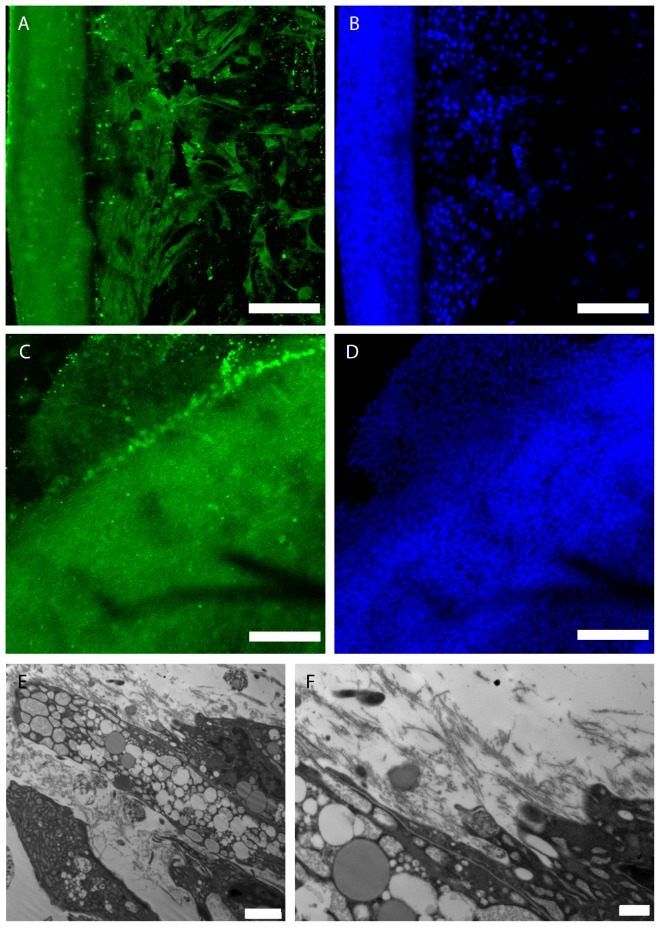
FACS characterization of human sfMPCs. Normal and OA CD90+ and CD90− subpopulations were assayed for expression of CD105, CD90, CD73, CD45 and CD11b prior to chondrogenic differentiation.

Human: Fresh synovial fluid was plated in untreated culture dishes and after 1–2 hrs at 37°C/5%CO_2_ culture media was added. sMPC culture media consisted of DMEM (Invitrogen # 11965), 10% FBS, 1% Pen/Strep, 1% Non-essential amino acids (NEAA), 0.2% Beta-mercaptoethanol (BME) (all Invitrogen, Carlsbad, CA). Once cells had adhered to the plastic and reached 30–40% confluence, the media was changed and the cells were allowed to reach 60–70% confluence. At this point the cells were dissociated and resuspended in Dulbecco's PBS (DPBS) at 1 million cells/ml. Primary labeled FACS antibodies (Table 1) to CD105, CD73, CD44, CD45, CD11b (All Becton, Dickinson and Company (BD), Franklin Lakes, NJ) were added to the suspension and the cells were sterilely sorted (University of Calgary, Flow Cytometry Core Facility). This CD105+, CD73+, CD44+, CD45−, CD11b− population accounted for 47.4%(+/−5.4%) of the total cells isolated from normal joints, and 67.2%(+/−3.1%) from joints with OA. Both normal and OA CD105+, CD73+, CD44+, CD45−, CD11b− cell populations were over 80% positive for CD90 before magnetic purification. The CD105+, CD73+, CD44+, CD45−, CD11b− cell population was returned to culture for 1 passage and then prepared for magnetic enrichment (an aliquot of this population was tested for multi-potency, with differentiation into fat, bone and cartilage assayed with oil red, alizarin red and Alcian blue respectively). Purified sfMPCs were dissociated and treated with anti-CD90 (BD) and then a magnetically labeled secondary (BD), and subsequently exposed to a magnetic field for enrichment following the manufacturer's instructions. CD90-positive and CD90-negative fractions were cultured for one additional passage and then induced to differentiate. An aliquot of the cells at this passage were analyzed using FACS to confirm the presence or absence of CD105, CD90, CD73, CD44, CD45, CD11b (Figure 6).

**Table 1:** Primary antibodies used for analyses.

Marker	Source	Identifies	Species	Cell target
CD105	Becton Dickinson	Endoglin	Human	MPC
CD73	Becton Dickinson	5′-nucleotidase	Human	MPC
CD44	Becton Dickinson	CD44	Human	MPC
CD45	Becton Dickinson	Protein tyrosine phosphatase, receptor type, C	Human	hematopoietic cells
CD11b	Becton Dickinson	Integrin alpha M	Human	hematopoietic cells
CD90	Becton Dickinson	Thy-1	Human/Canine	MPC
CD34	Becton Dickinson	CD34	Canine	hematopoietic cells
CD45	Aviva Systems Biology	Protein tyrosine phosphatase, receptor type, C	Canine	hematopoietic cells
Collagen 2	Hybridoma Bank, Iowa	Collagen 2	Human/Canine	Chondrocytes
Aggrecan	Santa Cruz	Aggrecan	Human/Canine	Chondrocytes

Canine: Fresh synovial fluid was plated in untreated culture dishes and after 1–2 hrs at 37°C/5%CO_2_ culture media was added. sMPC media (as above) was used. Once cells had adhered to the plastic and reached 30–40% confluence, the media was changed and the cells were allowed to reach 60–70% confluence. At this point the cells were dissociated and resuspended in Dulbecco's PBS (DPBS) at 1 million cells/ml. The total cell population was negatively purified using biotin-labeled antibodies to canine CD45 (Aviva Systems Biology, San Diego, CA) and CD34 (BD) following the manufacturer's instructions. This cell population was then selected for the CD90+ (BD) fraction using magnetic separation. CD90-positive and CD90-negative fractions were cultured for one additional passage and then induced to differentiate.

### Adipogenic Differentiation

sfMPCs were plated in triplicate (100,000 cells/well/24 well dish) and exposed to adipogenic differentiation media for 20 days. Differentiation media consisted of sMPC culture media with 0.5 mM isobutylmethylxanthine, 1 µM dexamethasone, 10 µM insulin, 200 µM indomethacin (all Sigma). Media was changed every three days during the 20 day differentiation period.

### Osteogenic Differentiation

sfMPCs were plated in triplicate (100,000 cells/well/24 well dish) and exposed to osteogenic differentiation media for 20 days. Differentiation media consisted of sMPC culture media with 0.1 µM dexamethasone and 50 µM ascorbate-2-phosphate. Media was changed every three days during the 20 day differentiation period.

### Chondrogenic Differentiation

sfMPCs were plated in triplicate (100,000 cells/well/24 well dish) and exposed to chondrogenic media for 14 days with or without micro-mass aggregation. Aggregation was achieved by placing 100,000 cells in a 1.5 ml sterile tube at 37°C overnight. Differentiation media consisted of sMPC culture media with 500 ng/mL BMP-2 (Peprotech, Rocky Hill, NJ), 10 ng/mL TGF-β3 (Peprotech), 10^−8^ M dexamethasone (Sigma, St. Louis, MO), 50 µg/mL ascorbic acid (Sigma), 40 µg/mL proline (Invitrogen), 100 µg/mL pyruvate (Sigma) and supplemented with insulin, transferrin, and selenium (Sigma). Media was changed every three days during the 14-day differentiation period.

### Analysis of Differentiation

Differentiation was assessed using quantitative RT-PCR (qRT-PCR) and immunofluorescence (IF).

qRT-PCR: RNA was collected using Trizol (Invitrogen) and converted to cDNA using a High Capacity cDNA kit (Applied Biosystems (ABS), Carlsbad, CA). The cDNA was probed using pre-validated Taqman™ primer-sets for human Sox9, Collagen 2, Aggrecan, Osterix (SP7), Osteocalcin, PPAR-gamma, Adiponectin, and canine Sox9, Collagen 2 and Aggrecan (Table 2) on an ABI 7900HT using 18S as the internal control and the ddCT method included within the ABI software to analyze results.

**Table 2:** qRT-PCR probes used for analysis.

Marker	Source	Cell Target
Sox 9	ABI (human and canine probes)	Chondrocytes
Collagen 2	ABI (human and canine probes)	Chondrocytes
Aggrecan	ABI (human and canine probes)	Chondrocytes
Osterix (SP7)	ABI (human)	Osteoblasts
Osteocalcin	ABI (human)	Osteoblasts
PPAR-gamma	ABI (human)	Adipocytes
Adiponectin	ABI (human)	Adipocytes

IF: Aggregates/tissues generated from sfMPCs were washed in PBS and fixed in 4% para-formaldehyde (PFA) in PBS at 4°C overnight. Aggregates were then permeabilized in 0.5% saponin (Sigma) in PBS at 4°C overnight, rinsed once in PBS, then blocked in 3% BSA at 4°C overnight. Primary antibodies (Collagen 2: Developmental Studies Hybridoma Bank, Iowa. Aggrecan: Santa Cruz, Santa Cruz, CA) (Table 1) were diluted 1∶50 in 3% BSA, added to the cell samples and incubated overnight at 4°C. The aggregates were then washed 3 times with PBS and blocked again overnight at 4°C. Following the block, the aggregates were incubated with an appropriate Alexa-fluor 488 secondary antibody (Molecular Probes, Carlsbad, CA) and Toto-3™ (Molecular Probes) overnight at 4°C. After incubation, the aggregates were washed thrice with PBS and mounted on slides with mountant (9∶1 glycerol∶PBS). Slides were analyzed using a Zeiss 510 confocal Microscope with 488, 568 and 633 nm filters. Images were prepared using Zeiss LSM image browsing software.

### Transmission Electron Microscopy

For transmission electron microscopy (TEM), pre-cartilaginous tissue-like aggregates were collected after differentiation, and then washed in PBS before fixation with 4% glutaraldehyde in Millonig's phosphate buffer for 1 hour at room temperature. Post-fixation was carried out in 2% OsO_4_ for 20 minutes. The tissues were then dehydrated in ethanol, and subsequently infiltrated with Polybed 812 resin (Polysciences). Polymerization was performed at 37°C for 24 hours. Silver-gray sections were cut with an ultramicrotome equipped with a diamond knife, and sections were stained with uranyl acetate and lead citrate, then examined in an H-7000 Hitachi electron microscope.

### Statistical Analysis

Each treatment (cell differentiation) and assay (IF, qRT-PCR) was performed in triplicate. Statistical analysis (ANOVA) was performed on qRT-PCR data using GraphPad Prism4 (GraphPad Software) and significance was set at p<0.05.
