# Correction: Synovial Fluid Progenitors Expressing CD90+ from Normal but Not Osteoarthritic Joints Undergo Chondrogenic Differentiation without Micro-Mass Culture

**DOI:** 10.1371/annotation/ace75296-7ec7-4193-ac91-5d68cfe5073e

**Published:** 2012-09-06

**Authors:** Roman J. Krawetz, Yiru Elizabeth Wu, Liam Martin, Jerome B. Rattner, John R. Matyas, David A. Hart

The legends for Figures 1-6 were incorrectly switched. The legend that appears with Figure 1 should be with Figure 2. The legend that appears with Figure 2 should be with Figure 3. The legend that appears with Figure 3 should be with Figure 4. The legend that appears with Figure 4 should be with Figure 5. The legend that appears with Figure 5 should be with Figure 6. The legend that appears with Figure 6 should be with Figure 1. The figure images appear in the correct order. 

